# Systemic inflammatory response drives post-cardiac arrest multi-organ injury via IL-17 signaling in murine and porcine models

**DOI:** 10.3389/fimmu.2026.1837813

**Published:** 2026-04-29

**Authors:** Yang Yan, Taiwei Chen, Ancai Yuan, Na Geng, Fang Wan, Peiliang Fang, Zhiqing Qiao, Zhaoling Wei, Jun Pu

**Affiliations:** 1 Department of Cardiology, Ren Ji Hospital, School of Medicine, Shanghai Jiao Tong University, Shanghai, China; 2 State Key Laboratory for Oncogenes and Related Genes, Shanghai Cancer Institute, Shanghai Jiao Tong University, Shanghai, China

**Keywords:** cardiac arrest, IL-17A, inflammation signaling, multi-organ injury, post-resuscitation syndrome, systemic inflammation

## Abstract

**Background:**

Systemic inflammation plays a central role in the development of multi-organ diseases. Post-cardiac arrest syndrome (PCAS) is increasingly recognized as a systemic inflammatory condition characterized by myocardial dysfunction, brain injury, and multi-organ dysfunction following resuscitation. Interleukin-17 signaling is an important proinflammatory pathway, but its specific role in the pathogenesis of PCAS remains unclear.

**Methods and results:**

Using transcriptomic profiling of both cardiac and cerebral tissues after cardiac arrest/cardiopulmonary resuscitation (CA/CPR), we found that myocardial dysfunction and brain injury were associated with early activation of the IL-17 signaling pathway during the post-resuscitation period. Further analysis of differentially expressed genes identified interleukin-17A (IL-17A) as a critical cytokine driving systemic inflammation and subsequent cardiac and cerebral dysfunction following CA/CPR. Consistently, plasma IL-17A levels were significantly elevated in patients who experienced CA after myocardial infarction compared with non-CA controls. Early inhibition of IL-17A with secukinumab in both murine and porcine models of CA/CPR significantly attenuated PCAS, including improved left ventricular ejection fraction, reduced neuronal apoptosis, and improved 72-hour survival (all *P* < 0.05), demonstrating multi-organ protective effects.

**Conclusions:**

These findings demonstrate that CA induces a systemic inflammatory response that drives heart–brain injury and multi-organ dysfunction, and IL-17A acts as an inflammatory mediator in this process. Targeting of IL-17A may represent a potential therapeutic strategy for inflammation-driven multi-organ injury after CA.

## 1 Introduction

Systemic inflammation has emerged as a central mechanism underlying the pathogenesis of various multi-organ diseases (1). Like other mediators of inter-organ crosstalk (2), persistent inflammation not only affects local tissue injury but also promotes inter-organ crosstalk, ultimately leading to multi-organ dysfunction and disease progression (3). Accumulating evidence suggests that systemic inflammatory responses play pivotal roles in the development of cardiovascular diseases, neurological disorders, and multi-organ failure syndromes (4–6).

Sudden cardiac arrest (CA) remains one of the leading causes of mortality worldwide (7). Despite advances in cardiopulmonary resuscitation (CPR), a substantial proportion of successfully resuscitated patients develop post-CA syndrome (PCAS) (8, 9), which is characterized by myocardial dysfunction, brain injury, systemic ischemia/reperfusion injury, and persistent precipitating pathology (10). Importantly, PCAS shares many pathophysiological features with systemic inflammatory response syndrome and sepsis-like conditions, including cytokine storm, immune activation, endothelial dysfunction, and multi-organ injury (11, 12). Therefore, PCAS can be considered a systemic inflammatory syndrome triggered by global ischemia/reperfusion injury.

Increasing evidence suggests that the inflammatory response following CA is not limited to the acute phase but may persist beyond the initial injury and contribute to prolonged organ dysfunction and poor long-term outcomes (13–15). Persistent inflammation after resuscitation may promote neuroinflammation, exacerbate myocardial injury, and contribute to prolonged organ dysfunction, indicating that PCAS may represent a dynamic inflammatory process evolving from systemic inflammation to persistent inflammatory injury (16, 17). However, the key inflammatory mediators that initiate systemic inflammation after resuscitation remain largely unclear.

In the present study, by utilizing transcriptomic profiling of heart and brain tissues after CA/CPR, we identified IL-17 signaling pathway as an early activated inflammatory pathway and IL-17A as a critical cytokine driving systemic inflammation and multi-organ injury. Early pharmacological inhibition of IL-17A alleviated myocardial dysfunction, brain injury, and improved survival in both murine and porcine models. These findings suggest that systemic inflammatory signaling mediated by IL-17 plays a key role in post-CA multi-organ dysfunction.

## 2 Materials and methods

### 2.1 Reagents

Antibodies against Bax, Bcl-2, Caspase3 and cleaved-Caspase3 were obtained from Cell Signaling Technology (MA, USA). Antibodies against GAPDH were purchased from ShareBio (Shanghai, China). Peroxidase conjugated secondary antibodies were obtained from Jackson ImmunoResearch Laboratories (PA, USA). The BCA protein assay kit was purchased from Thermo Fisher (CA, USA).

### 2.2 Human studies

All study patient data were obtained from the resources of the EARLY-MYO-CMR (Early Assessment of Myocardial Tissue Characteristics by CMR in STEMI) registry, which was a prospective, multicenter registry of patients with acute ST-elevation myocardial infarction (STEMI) who have undergone CMR imaging (NCT03768453) (18–22). The study was approved by the Ethics Committee of Renji Hospital (approved documents: KY2025-005-B) and conducted according to the criteria set by the Declaration of Helsinki (2013). Informed consent was obtained from all participants. Criteria for participant eligibility and exclusion were described in our previous studies (18). We analyzed 26 consecutive survivors with sudden CA resulted from STEMI who underwent CPR. Non-CA/CPR control patients from the EARLY-MYO-CMR were matched using propensity score-matched analysis (PSM) in baseline characteristics. The clinical characteristics of acute STEMI patients suffering from CA and their non-CA PSM-matched controls are shown in **Supplementary Table 1**.

### 2.3 Animal studies

The protocols for double-blind and randomized animal experiments were approved by the Institute’s Animal Ethics Committee of Shanghai Jiao Tong University, School of Medicine, Renji Hospital (approved documents: RJ2024-227B and RJ2024-210B) and adhered to the NIH Guidelines for the Care and Use of Laboratory Animals. C57BL/6J mice (8–10 weeks old, weighing 15–20 g) and Yorkshire farm-bred pigs (3–4 months old, weighing 30–40 kg) of both sexes were included in this study. Animals were randomly assigned to experimental groups using simple randomization, with comparable age and sex distribution across groups. Animals were housed at 24 °C ± 2 °C with a humidity of 40% ± 5% under a 12-hour light/dark cycle with free access to food and water. The total number of animals used in this study was 80 mice and 12 pigs. Prior to the experiments, all animals were assessed for general health status by trained veterinarians in accordance with NIH Guidelines for the Care and Use of Laboratory Animals.

#### 2.3.1 Establishment of a modified mouse model of CA/CPR

**Figure 1:**
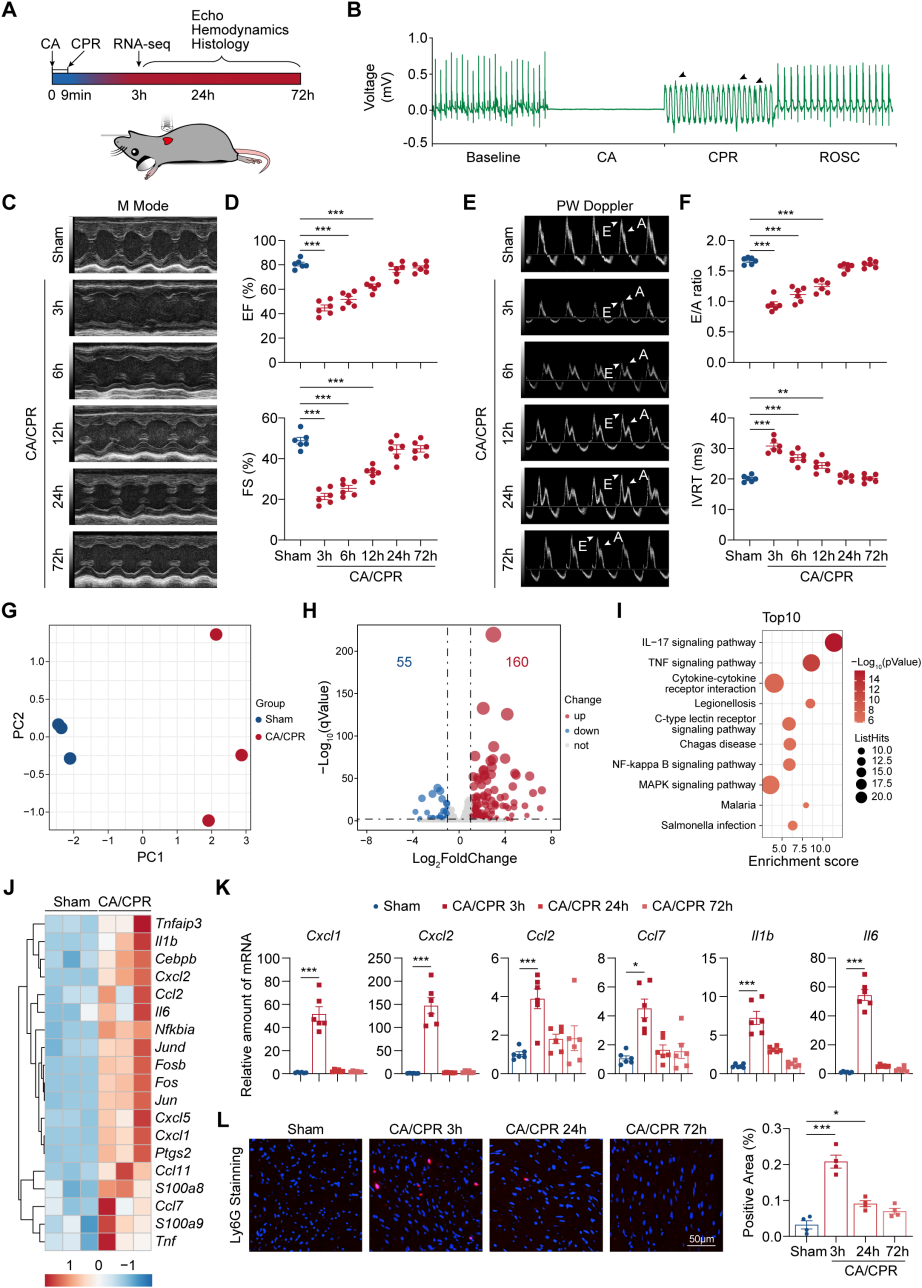
Early activation of IL-17 signaling pathway in CA/CPR-induced myocardial dysfunction. **(A)** Schematic protocol for CA/CPR murine model establishment and subsequent analysis. **(B)** Representative curve of ECG at stages of Baseline, CA, CPR, and ROSC. Black arrows indicated spontaneous contraction during CPR. **(C)** Representative M-mode echocardiograms in indicated groups. **(D)** Echocardiographic measurements of EF and FS in indicated groups (n = 6). Data were analyzed by one-way ANOVA followed by Bonferroni’s *post hoc* test. ***P < 0.001. **(E)** Representative PW Doppler-mode echocardiograms in indicated groups. White arrows indicated E and A waves. **(F)** Echocardiographic measurements of E/A ratio and IVRT in indicated groups (n = 6). Data were analyzed by one-way ANOVA followed by Bonferroni’s *post hoc* test. **P < 0.01; ***P < 0.001. **(G)** Principal components analysis of genes expressions of heart tissues from Sham and CA/CPR groups at 3 h post-resuscitation (female mice, n = 3). **(H)** The volcano plot showing the differentially expressed genes in CA/CPR versus Sham groups at 3 h post-resuscitation (female mice, n = 3). **(I)** The top 10 KEGG pathways of differentially expressed genes. **(J)** Heatmap of differential expressed genes enriched in IL-17 signaling pathway (n = 3). **(K)** The expressions of chemokines and interleukins involved in IL-17 pathway were detected by RT-qPCR in heart tissues harvested from Sham and CA/CPR groups at indicated times (n = 6). Data were analyzed by one-way ANOVA followed by Bonferroni’s *post hoc* test. ***P < 0.001. **(L)** Left panel: Neutrophils infiltration in heart tissues was measured by immunofluorescence staining of Ly6G at indicated times post-Sham and CA/CPR (n = 4, scale bar = 50 μm). Right panel: Quantification of immunofluorescence staining of Ly6G presented in the upper panel (n = 4). Data were analyzed by one-way ANOVA followed by Bonferroni’s *post hoc* test. *P < 0.05; ***P < 0.001. CA, indicates cardiac arrest; CPR, cardiopulmonary resuscitation; ECG, electrocardiogram; EF, ejection fraction; FS, fractional shortening; IVRT, isovolumic relaxation time; KEGG, Kyoto Encyclopedia Genes and Genomes; PW, pulsed wave; ROSC, return of spontaneous circulation.

A modified mouse model of CA/CPR with high reproducibility was established based on a mini-sewing machine-induced automatic chest compression procedure (**Figures 1A, B**). Mice were anesthetized in an anesthesia induction chamber filled with 2.0% isoflurane/oxygen. A tracheal catheter was then quickly inserted and attached to a micro-ventilator (MiniVent 845; Harvard Apparatus, MA, USA) for maintenance of anesthesia (1.0% isoflurane/oxygen) and later mechanical ventilation. Electrocardiogram (ECG) rechargeable telemetry system (Kardiotek, Shanghai, China) was prepared for identification of successful induction of CA and ROSC. For fluid administration, heparinized PE tube (SDR Scientific, Sydney, Australia) was placed into the right jugular vein. Hemodynamic recording was achieved by catheterization using a 1.4 Frenchmicro-manometer (Millar Instruments, TX, USA) through femoral artery. Rectal temperature was monitored and maintained at 37.0 ± 0.5 °C during surgery with a warming pad.

CA was induced by injection of potassium chloride (KCl, 0.08 mg/g) through jugular vein, with ventilator removed from tracheal catheter. Successful induction of CA was achieved as indicated by cessation of cardiac electrical activity shown in ECG. After 9 min of CA, the mechanical ventilator was connected and a modified chest compression procedure was performed at the initiation of CPR process. The automatic mechanical chest compression system is running at a rate of 350/min, which imitates the rate of compression procedure delivered by human finger that described previously (23, 24). To stimulate the spontaneous myocardial contraction, epinephrine was infused with a dosage of 0.6 µg/g after initiation of CPR. Chest compressions were sustained until ROSC was achieved or 5 min after unsuccessful CPR. ROSC was defined as the return of sinus rhythm with heart rate > 200 beats per minute (BPM) and mean arterial pressure (MAP) > 40 mmHg lasting for at least 10 seconds. Mice were weaned form mechanical ventilation 30 min after ROSC and were then kept in a cage maintained at 32 °C by a warming lamp for further 3 hours. Thereafter, mice received intraperitoneal injection of 0.5 ml glucose saline for prevention of dehydration and returned to the regular cages (24 °C ± 2 °C) for further studies. Neurological scoring in each group was determined using a grading system for mice as previously reported (25).

For IL-17A inhibition, secukinumab (Selleck Chemicals, Shanghai, China), an IL-17A monoclonal antibody, was selected and administered intraperitoneally at a dose of 10 mg/kg based on our pilot study and previous murine models (26, 27), using IgG1 as an isotype control. At the end of the experiment, mice were euthanized by overdose of isoflurane (3% for inhalation), followed by cervical dislocation after confirmation of deep anesthesia.

#### 2.3.2 Establishment of a porcine model of CA/CPR

Pigs were anesthetized via intramuscular administration of tiletamine (2.5 mg/kg), zolazepam (2.5 mg/kg) and xylazine (10 mg/kg). Following induction of anesthesia, a tracheal catheter was quickly inserted and attached to an anesthesia machine (Veta 5; Mindray, Shenzhen, China) for maintenance of anesthesia (2.0% isoflurane/oxygen) and mechanical ventilation. Mechanical ventilation was administered with a tidal volume of 10 ml/kg at a frequency of 12 breaths/min. Monitor/defibrillator (M3535A; Philips Medical Systems, USA) was prepared for defibrillation and identification of successful CA induction and ROSC. Under ultrasound guidance, the femoral vein was cannulated using a 7 Fr introducer catheter (Avanti, USA). A balloon-tipped, bipolar pacing catheter (Medtronic, USA) was advanced into the right ventricle and connected to a single-chamber temporary pacemaker (Medtronic, USA). Similarly, the femoral artery was cannulated using a 6 Fr introducer catheter (TERUMO, Japan) under ultrasound guidance for continuous invasive arterial blood pressure monitoring. Baseline hemodynamic measurements and blood samples were obtained prior to the induction of CA.

CA was induced by delivery of a 9 V direct current via the pacing catheter, as confirmed by the onset of ventricular fibrillation (VF) on ECG. Mechanical ventilation and isoflurane administration were discontinued immediately following CA induction. After 6 min of CA, mechanical ventilation was resumed and chest compressions were performed using a mechanical chest compression device (LUCAS, Stryker, USA) at a rate of 100 compressions per minute. The first defibrillation attempt and intravenous administration of epinephrine (0.02 mg/kg) were performed after 2 min of CPR. Additional defibrillation was delivered every 2 min and epinephrine was administered every 4 min in the absence of ROSC. CPR was sustained until ROSC was achieved or 30 min after unsuccessful CPR. ROSC was defined as the sustained restoration of sinus rhythm with a MAP > 60 mmHg lasting for at least 2 min. For IL-17A inhibition, secukinumab was administered intravenously at a dose of 300 mg. The 300 mg dose was selected based on the clinically approved dosing regimen for secukinumab in human inflammatory diseases and adjusted for the body weight range of the porcine subjects (approximately 30–40 kg), yielding a weight-based dose of approximately 7.5–10 mg/kg, which is consistent with the murine dosing strategy (10 mg/kg) and prior preclinical studies. At the end of the experiment, pigs were euthanized under deep anesthesia by rapid intravenous injection of 10% potassium chloride (150mg/kg) via the ear vein.

### 2.4 Echocardiography for animals

For mice, cardiac systolic and diastolic function were measured using echocardiography (Vevo 2100; VisualSonics, Toronto, Canada) with a 40 MHz MX transducer (VisualSonics) (28). For pigs, hearts were visualized by echocardiography using a Vivid Q ultrasound system from GE healthcare (General Electric, Chicago, IL, USA) with a 1.9–4 MHz transducer. Animals were anesthetized with isoflurane (2% induction, 1% maintenance) and placed supine during operation. Left parasternal long axes and cardiac four-chamber view images were recorded. Systolic function parameters involving ejection fraction (LVEF) and fractional shortening (LVFS), and diastolic function involving E/A ratio were calculated.

### 2.5 RNA-sequencing and data analysis

The resuscitated heart samples at indicated times were collected and total RNA was isolated using the RNAiso Plus reagent (TAKARA, Japan). RNA integrity was evaluated using the Agilent 2100 Bioanalyzer (Agilent Technologies, Santa Clara, CA, USA). The samples with RNA Integrity Number (RIN) ≥ 7 were selected for subsequent analysis. The libraries prepared from three biological replicates for each group were constructed using TruSeq Stranded mRNA LTSample Prep Kit (Illumina, San Diego, CA, USA). Then these libraries were sequenced on the Illumina sequencing platform Illumina HiSeq X Ten and 150 bp paired-end reads were generated. Raw reads were processed using Trimmomatic (29). Clean reads were aligned against the mouse genome (GRCm38) using hisat2 (30) and the read counts of genes were obtained by htseq-count (31). Differential expression analysis was performed using the DESeq2 R package (v1.26.0). KEGG pathway enrichment analysis was conducted using the clusterProfiler R package based on the hypergeometric distribution, with the KEGG database accessed in September 2025. Adjust *P* value < 0.05 and FoldChange > 2 or FoldChange < 0.5 was set as the threshold for significantly differential expression.

### 2.6 Real-time quantitative polymerase chain reaction

Total RNA was extracted from heart tissues with the RNAiso Plus reagent (TAKARA, Japan). Reverse transcription was carried out using HiScript III RT SuperMix for qPCR with gDNA wiper (Vazyme Biotech, Nanjing, China). RT-qPCR was conducted using ChamQ Universal SYBR qPCR Master Mix (Vazyme Biotech, Nanjing, China) in LightCycler 480 System (Roche Applied Science, Indianapolis, IN, USA). Primers were obtained from Sangon Biotech Co., Ltd. (Shanghai, China). Primer sequences are listed in **Supplementary Table 2**.

### 2.7 Western blot and data analysis

Total protein from tissues were extracted using lysis buffer (Boster Biological Technology Co., Ltd., Pleasanton, CA, USA) and protein concentrations were detected using the Pierce BCA Protein Assay Kit (Thermo Scientific, Carlsbad, CA, USA). Proteins were separated using 10% SDS-PAGE and then transferred to PVDF membranes (Millipore, Billerica, MA, USA). Before adding indicated primary antibodies, membranes were blocked in 5% non-fat milk Tris-buffer for 1 h at room temperature. Protein bands were then detected by incubating with horseradish peroxidase-conjugated secondary antibodies and enhanced chemiluminescence reagent (ShareBio, Shanghai, China). The protein expression levels were quantified by detecting densitometric values of protein bands using Image J software (Version 1.48v, NIH, Bethesda, MD, USA) and normalized to loading controls. The uncut blot is shown in **Supplementary Figure 3**.

### 2.8 Immunofluorescence staining

For immunofluorescence staining analysis, the heart tissues and brain tissues were collected and embedded in optimum cutting temperature compound (SAKURA, USA) and serial 5 mm-thick cryosections of tissues were prepared. To assess the neutrophils infiltration in myocardium, frozen slides were fixed, permeabilized and then incubated with anti-Ly6G antibody (Santa Cruz Biotechnology, Texas, USA) and corresponding secondary antibody. Slides were finally counterstained with DAPI and imaged using a fluorescence microscope (Leica DM3000B, Germany). The positive staining area was quantified using Image J software (Version 1.48v, NIH, Bethesda, MD, USA).

### 2.9 Measurements of the IL-17 levels by enzyme-linked immunosorbent assay

Serum levels of IL-17A, E, F in mice were detected using corresponding ELISA Kit (MultiSciences, Hangzhou, China), and levels of IL-17B, C, D were measured by corresponding ELISA Kit (Raybiotech, GA, USA). Plasma levels of IL-17A in patients were detected using Human IL-17A ELISA Kit (MultiSciences, Hangzhou, China). Optical densities of samples were determined at 450 nm using Multiskan GO Microplate Spectrophotometer (Thermo Fisher Scientific, MA, USA). Analyses were performed according to the manufacturers’ instructions for each ELISA kit.

### 2.10 Measurements of blood-brain barrier permeability

Determination of blood-brain barrier permeability was conducted 24 h after CA in mice. Briefly, mice were anesthetized by 2% isoflurane and injected with 2% Evan’s blue at a dose of 4 ml/kg through tail vein. After 4 h circulation of Evan’s blue, mice were perfused with ice-cold PBS and sacrificed. The brain was collected, homogenized in 1 ml of 50% trichloroacetic acid (Sigma-Aldrich, Saint Louis, MI, USA), and centrifuged for 20 min at 20,000 g. Evan’s blue dye was measured by Cytation 3 Cell Imaging Multi-Mode Reader (Winooski, Vermont, USA) with excitation at 620 nm and emission at 680 nm. Evan’s blue dye extravasation was expressed as microgram per gram of brain tissue (μg/g).

### 2.11 *In-situ* detection of neuronal apoptosis in brain

*In-situ* detection of neuronal apoptosis was detected by terminal deoxynucleotidyl transferase dUTP nick-end labeling (TUNEL) staining using an *In Situ* Cell Death Detection Kit (Roche Diagnostics). Neurons were labeled by anti-NeuN antibody (Proteintech, Wuhan, China), apoptotic nuclei were identified by green fluorescein staining, and total nuclei were stained by DAPI. Apoptotic index was expressed as the percentage of apoptotic nuclei over the total nuclei.

### 2.12 Statistical analysis

Data are presented as means ± SEM. Comparisons between the two groups were performed using unpaired Student’s *t*-test. Multiple groups were performed using one-way ANOVA or two-way ANOVA with Bonferroni *post hoc* analysis. Comparisons of survival rate between groups were evaluated by the Kaplan-Meier method with a log-rank (Mantel-Cox) test. Bivariate comparisons of categorical variables were performed with the Fisher exact test. Comparisons of neurological score between two groups were assessed by Mann–Whitney *U* test. Because human sudden CA studies depend on collected data and cannot be randomized, a PSM analysis was used to match the baseline characteristics of 2 groups for survivors from sudden CA and non-CA controls. PSM is a widely used statistical technique to adjust for differences in baseline characteristics in studies incapable of randomization (32, 33). Statistical analysis was conducted with GraphPad Pro Prism 8.01 (GraphPad, San Diego, CA, USA) and R version 3.6.0 (R Foundation for Statistical Computing). Statistical significance was determined as *P* value < 0.05.

## 3 Results

### 3.1 Early activation of IL-17 signaling pathway in CA/CPR-induced myocardial dysfunction

To investigate the gene expression pattern in CA/CPR-induced myocardial injury, we first established a modified murine CA/CPR model using an automatic mechanical chest compression system (**Figure 1A**). Electrocardiogram (ECG) recordings confirmed the successful induction of CA and the subsequent effective CPR procedure (**Figure 1B**). Echocardiographic and hemodynamic analysis revealed that myocardial dysfunction was most severe at 3 h post-resuscitation and gradually resolved within 72 h (**Figures 1C–F**; **Supplementary Figures 1A, B**). To elucidate the molecular mechanisms underlying this acute cardiac dysfunction, we performed transcriptomic analysis on heart tissues collected at early stage of post-CA/CPR (3 h) and compared them to sham-operated controls. Principal components analysis (PCA) revealed a distinct heterogeneity of transcriptional profiles between post-resuscitation and Sham groups (**Figure 1G**). Volcano plot showed DEGs in CA/CPR group versus Sham group, with 160 upregulated genes and 55 downregulated genes (**Figure 1H**). Next, we employed KEGG enrichment analysis to further dictate the critical processes involved in myocardial dysfunction post-resuscitation. Interrogation of DEGs by KEGG analysis identified a typical transcriptomic signature for proinflammatory process including IL-17 signaling pathway, TNF signaling pathway and cytokine-cytokine receptor interaction in early post-CA/CPR period (**Figure 1I**). We observed that the early alteration of enriched genes within the top 1 ranked pathway (IL-17 signaling pathway, **Supplementary Figure 2A**) was significantly reversed in a time-dependent manner after resuscitation (**Figures 1J, K**). Many of these genes have been previously implicated in ischemic insult including chemokines (*Cxcl1*, *Cxcl2*, *Ccl2*, *Ccl7*) and interleukins (*Il1b*, *Il6*), which contributed to leucocytes like neutrophils infiltration and further inflammatory responses (34–36). As expected, we observed that the recruitment of neutrophils in heart tissues was significantly reversed in a time-dependent manner after resuscitation, as evidenced by immunofluorescence staining of Ly6G (**Figure 1L**). Taken together, transcriptomic profiling of heart tissues reveals a significant inflammatory response at early stage of post-CA resuscitation and gradually recovered within 72 h after CA.

### 3.2 Early activation of IL-17 signaling pathway in CA/CPR-induced brain injuries

**Figure 2:**
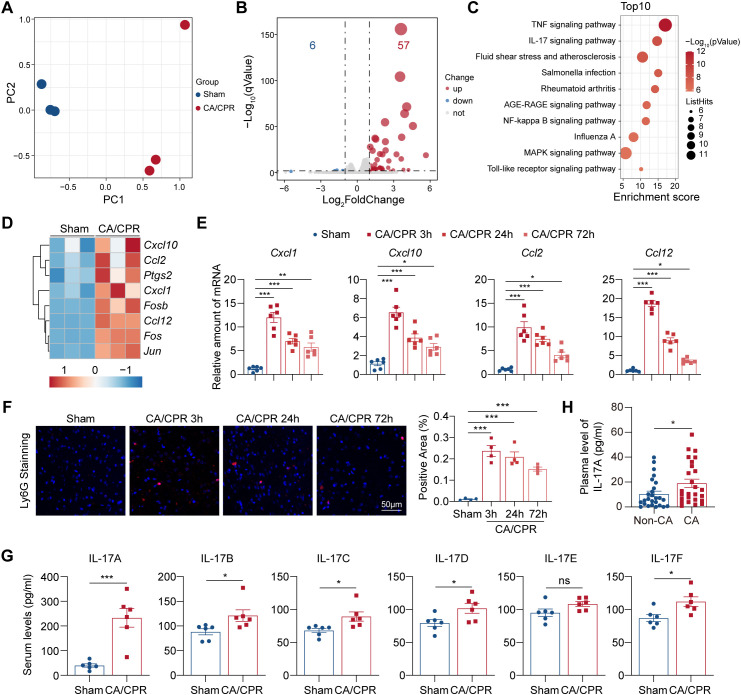
Early activation of IL-17 signaling pathway in CA/CPR-induced brain injuries. **(A)** Principal components analysis for genes expressions of whole brain tissues from Sham and CA/CPR groups at 3 h post-resuscitation (female mice, n = 3). **(B)** The volcano plot showing the differentially expressed genes in CA/CPR versus Sham groups at 3 h post-resuscitation (female mice, n = 3). **(C)** The top 10 KEGG pathways of differentially expressed genes. **(D)** Heatmap of differential expressed genes enriched in IL-17 signaling pathway (n = 3). **(E)** The expressions of chemokines involved in IL-17 pathway were detected by RT-qPCR in brain tissues harvested from Sham and CA/CPR groups at indicated times (n = 6). Data were analyzed by one-way ANOVA followed by Bonferroni’s *post hoc* test. *P < 0.05; **P < 0.01; ***P < 0.001. **(F)** Left panel: Neutrophils infiltration in cerebral cortex was measured by immunofluorescence staining of Ly6G at indicated times post-Sham and CA/CPR (n = 4, scale bar = 50 μm). Right panel: Quantification of immunofluorescence staining of Ly6G presented in the left panel (n = 4). Data were analyzed by one-way ANOVA followed by Bonferroni’s *post hoc* test. ***P < 0.001. **(G)** Serum levels of IL-17A, IL-17B, IL-17C, IL-17D, IL-17E, IL-17F in Sham and CA/CPR groups at 3 h post-resuscitation (n = 6). Data were analyzed by Student *t* test. *P < 0.05; ***P < 0.001; ns = not significant. **(H)** Plasma levels of IL-17A in survivors with sudden CA resulted from STEMI who underwent CPR and their non-CA PSM-matched controls (n = 26 per group). Data were analyzed by Mann–Whitney *U* test. *P < 0.05. CA indicates cardiac arrest; CPR, cardiopulmonary resuscitation; KEGG, Kyoto Encyclopedia Genes and Genomes; PSM, propensity score-matched; RT-qPCR, real-time quantitative polymerase chain reaction.

To explore the mechanism mediating CA/CPR-induced brain injuries, transcriptome analysis was also conducted in brain tissues collected from CA/CPR-treated and sham-operated mice. PCA revealed a clear separation of CA/CPR group from Sham group (**Figure 2A**). Volcano plot indicated a total of 63 DEGs (57 upregulated, 6 downregulated) in CA/CPR group versus Sham group (**Figure 2B**), with most genes enriched in TNF and IL-17 signaling pathways in early post-CA/CPR period (**Figures 2C, D**; **Supplementary Figure 2B**). Different from the results observed in heart, we noted that the upregulated genes involved in IL-17 pathway were partially reversed but sustained in a time-dependent manner after resuscitation (**Figures 2D, E**). Concomitantly, Ly6G immunofluorescence staining showed that the recruitment of neutrophils in brain tissues was also partially reversed but retained in a time-dependent manner after resuscitation (**Figure 2F**). These findings suggest that the early activation of inflammatory response in brain tissues is sustained, at least in part, lasting for 72 h during post-resuscitation period.

Combined observation from transcriptomic profiling of both heart and brain tissues identified that IL-17 signaling pathway was the most significant changed pathway after CA/CPR (**Figures 1I**, **2C**). Notably, IL-17A, the prototypical cytokine of IL-17 family (37), served as a major activator driving expressions of DEGs in both heart and brain tissues in response to CA/CPR (**Supplementary Figures 2A, B**). Further investigation of serum IL-17 isoforms including IL-17A, B, C, D, E, and F identified IL-17A as the cytokine with highest fold change post-resuscitation (**Figure 2G**). Moreover, the blood levels of IL-17A were found to be higher in patients suffering from CA after myocardial infarction relative to their non-CA controls (**Figure 2H**). Collectively, these data indicate that the IL-17 signaling pathway, driven by IL-17A, is significantly upregulated in the setting of CA/CPR.

### 3.3 Early inhibition of IL-17A suppresses myocardial inflammation and improves cardiac function in a murine CA/CPR model

**Figure 3:**
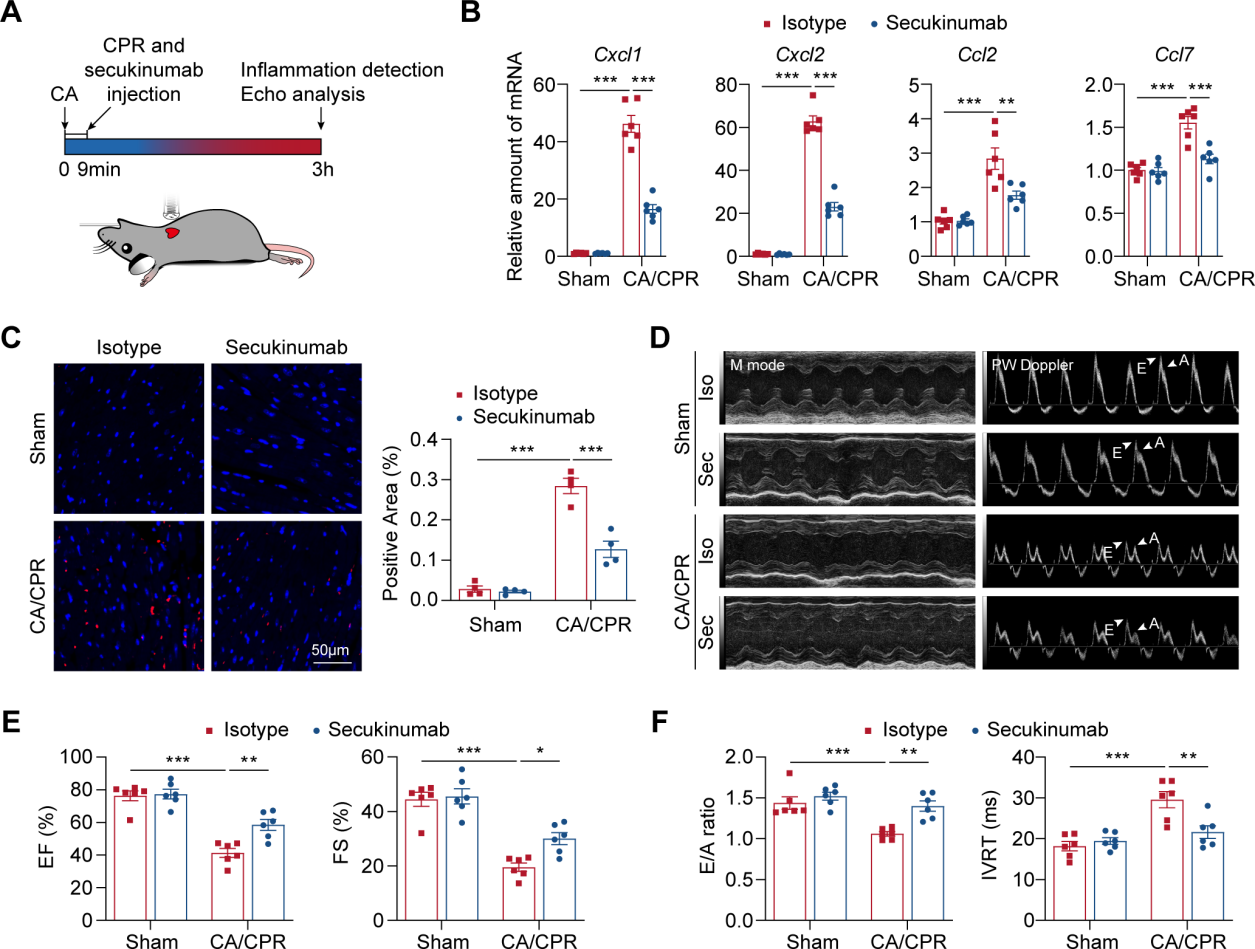
Early inhibition of IL-17A suppresses myocardial inflammation and improves cardiac function in CA/CPR mice. **(A)** Schematic representation of the experiment design. **(B)** Relative mRNA expression of chemokines in indicated groups after 3 h post-CA/CPR (n = 6). Data were analyzed by 2-way ANOVA followed by the Bonferroni *post hoc* test. **P < 0.01; ***P < 0.001. **(C)** Left panel: Representative immunofluorescence images of Ly6G staining in indicated groups after 3 h post-CA/CPR (n = 4, scale bar = 50 μm). Right panel: Quantification of immunofluorescence staining presented in the left panel (n = 4). Data were analyzed by 2-way ANOVA followed by Bonferroni’s *post hoc* test. ***P < 0.001. **(D)** Representative M-mode and PW Doppler-mode echocardiograms of mice in indicated groups after 3 h post-CA/CPR. **(E, F)** Quantification of EF, FS, E/A ratio and IVRT in indicated groups (n = 6). White arrows indicated E and A waves. Data were analyzed by 2-way ANOVA followed by Bonferroni’s *post hoc* test. *P < 0.05; **P < 0.01; ***P < 0.001. CA indicates cardiac arrest; CPR, cardiopulmonary resuscitation; EF, ejection fraction; FS, fractional shortening; IL-17, interleukin-17; IVRT, isovolumic relaxation time.

Given the specific inflammatory changes caused by activation of IL-17 pathway at early post-CA/CPR stage, we hypothesized that inhibition of IL-17 signaling may affect myocardial inflammation and dysfunction after CA/CPR. Inhibition of IL-17A was achieved by intraperitoneal injection of secukinumab, a monoclonal antibody targeting IL-17A, at the initiation of CPR procedure (**Figure 3A**). Early secukinumab administration resulted in a remarkable reduction of *Cxcl1*, *Cxcl2*, *Ccl2* and *Ccl7* levels compared with isotype control after resuscitation (**Figure 3B**). In parallel, immunostaining of heart tissues for Ly6G confirmed a diminished neutrophil infiltration in secukinumab-treated compared with isotype-treated mice (**Figure 3C**). These results clearly show that IL-17A inhibition suppresses myocardial inflammation at early post-CA/CPR stage. We next examined whether IL-17A inhibition altered myocardial dysfunction in CA/CPR. As shown in **Figures 3D–F**, echocardiography revealed that IL-17A inhibition significantly improved cardiac function after CA/CPR compared with isotype control, as evidenced by increased EF and FS. Notably, diastolic function was also significantly improved, as reflected by normalization of E/A ratio and IVRT. Collectively, these findings indicate that early inhibition of IL-17A by secukinumab alleviates myocardial inflammation and improves cardiac dysfunction after CA/CPR challenge.

### 3.4 Early inhibition of IL-17A mitigates brain injury and improves neurological outcomes in a murine CA/CPR model

**Figure 4:**
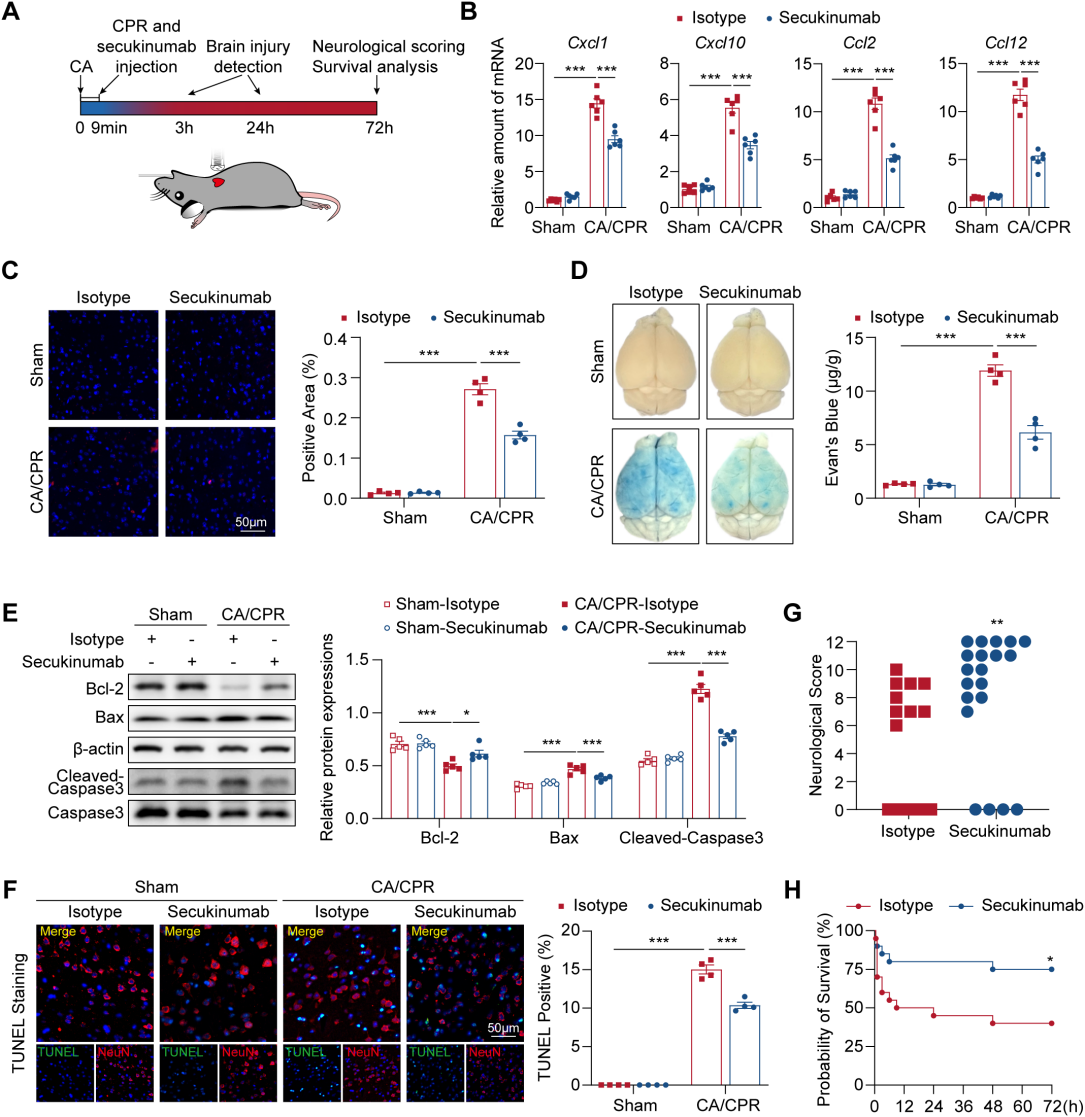
Early inhibition of IL-17A mitigates brain injury and improves neurological outcomes in CA/CPR mice. **(A)** Schematic representation of the experiment design. **(B)** Relative mRNA expression of chemokines in indicated groups after 3 h post-CA/CPR (n = 6). Data were analyzed by 2-way ANOVA followed by the Bonferroni *post hoc* test. ***P < 0.001. **(C)** Left panel: Representative Ly6G staining immunofluorescence images of cerebral cortex in indicated groups after 3 h post-CA/CPR (n = 4, scale bar = 50 μm). Right panel: Quantification of immunofluorescence staining presented in the left panel (n = 4). Data were analyzed by 2-way ANOVA followed by Bonferroni’s *post hoc* test. ***P < 0.001. **(D)** Left panel: Representative whole brain photographs of indicated groups after 24 h post-CA/CPR. Right panel: Evaluation of the extrusion levels of Evan’s blue in the brains (n = 4). Data were analyzed by 2-way ANOVA followed by the Bonferroni *post hoc* test. ***P < 0.001. **(E)** Left panel: Expression levels of apoptosis-related proteins in cerebral cortex from indicated groups after 24 h post-CA/CPR (n = 5). Right panel: Quantification of protein expressions in indicated groups. Data were analyzed by 2-way ANOVA followed by the Bonferroni *post hoc* test. *P < 0.05; ***P < 0.001. **(F)** Left panel: Representative images of neuron apoptosis in cerebral cortex were visualized by TUNEL staining (n = 4, scale bar = 50 μm). Right panel: Quantification of immunofluorescence staining presented in the left panel (n = 4). Data were analyzed by 2-way ANOVA followed by Bonferroni’s *post hoc* test. ***P < 0.001. **(G)** Neurological scoring of mice after 24 h post-CA/CPR in indicated groups. Of note, dead mice (11 in Isotype group and 4 in Secukinumab group) were also shown in the figure (score = 0) but excluded from statistical analysis. Data were analyzed by Mann–Whitney *U* test. **P < 0.01. **(H)** Survival curves in indicated groups (n = 20 per group). Survival data were analyzed by the Kaplan-Meier method and compared using log-rank tests. *P < 0.05. CA indicates cardiac arrest; CPR, cardiopulmonary resuscitation; IL-17, interleukin-17; TUNEL, terminal deoxynucleotidyl transferase dUTP nick-end labeling.

To further understand the impact of IL-17A inhibition at the initiation of CPR on brain injury and neurological outcomes (**Figure 4A**), we first detected chemokines expressions of brain tissues involved in IL-17 signaling pathway in CA/CPR and Sham groups. Secukinumab administration significantly reduced mRNA levels of *Cxcl1*, *Cxcl10*, *Ccl2* and *Ccl12* compared with isotype control after resuscitation (**Figure 4B**). In addition, immunostaining of brain tissues for Ly6G showed a reduced neutrophil infiltration in secukinumab-treated compared with isotype-treated mice (**Figure 4C**). Impairment of permeability of blood brain barrier (BBB) and neuronal apoptosis after CA/CPR are considered to be associated with poor neurological outcomes and low survival rate (38, 39). Thus, we further detected whether inhibition of IL-17A reversed these changes. We found that increased permeability of BBB was partially reversed in secukinumab-treated mice compared to isotype control, as indicated by the reduction of Evan’s blue extrusion in brain tissues (**Figure 4D**). We also showed that inhibition of IL-17A attenuated CA-induced activation of Caspase-3, which was concomitant with the reduction of Bax and partially restored Bcl-2 expressions (**Figure 4E**). Particularly, TUNEL staining revealed that apoptotic neurons were decreased after inhibition of IL-17A (**Figure 4F**), suggesting a mitigated neuronal apoptosis after resuscitation. Concomitantly, secukinumab improved neurological score after 72 h post-resuscitation (**Figure 4G**). Finally, early inhibition of IL-17A by secukinumab at the initiation of CPR significantly improved survival rates compared with isotype control (**Figure 4H**). Notably, late inhibition of IL-17A, as exerted by administration of secukinumab in 3 h or 24 h post-resuscitation period, showed no significant difference in survival rates among groups (**Supplementary Figures 3A, B**). Taken together, these results suggest that early inhibition of IL-17A mitigates brain injury and improves neurological outcomes and survival after CA/CPR challenge.

### 3.5 Early inhibition of IL-17A suppresses myocardial inflammation and improves cardiac function in a porcine CA/CPR model

**Figure 5:**
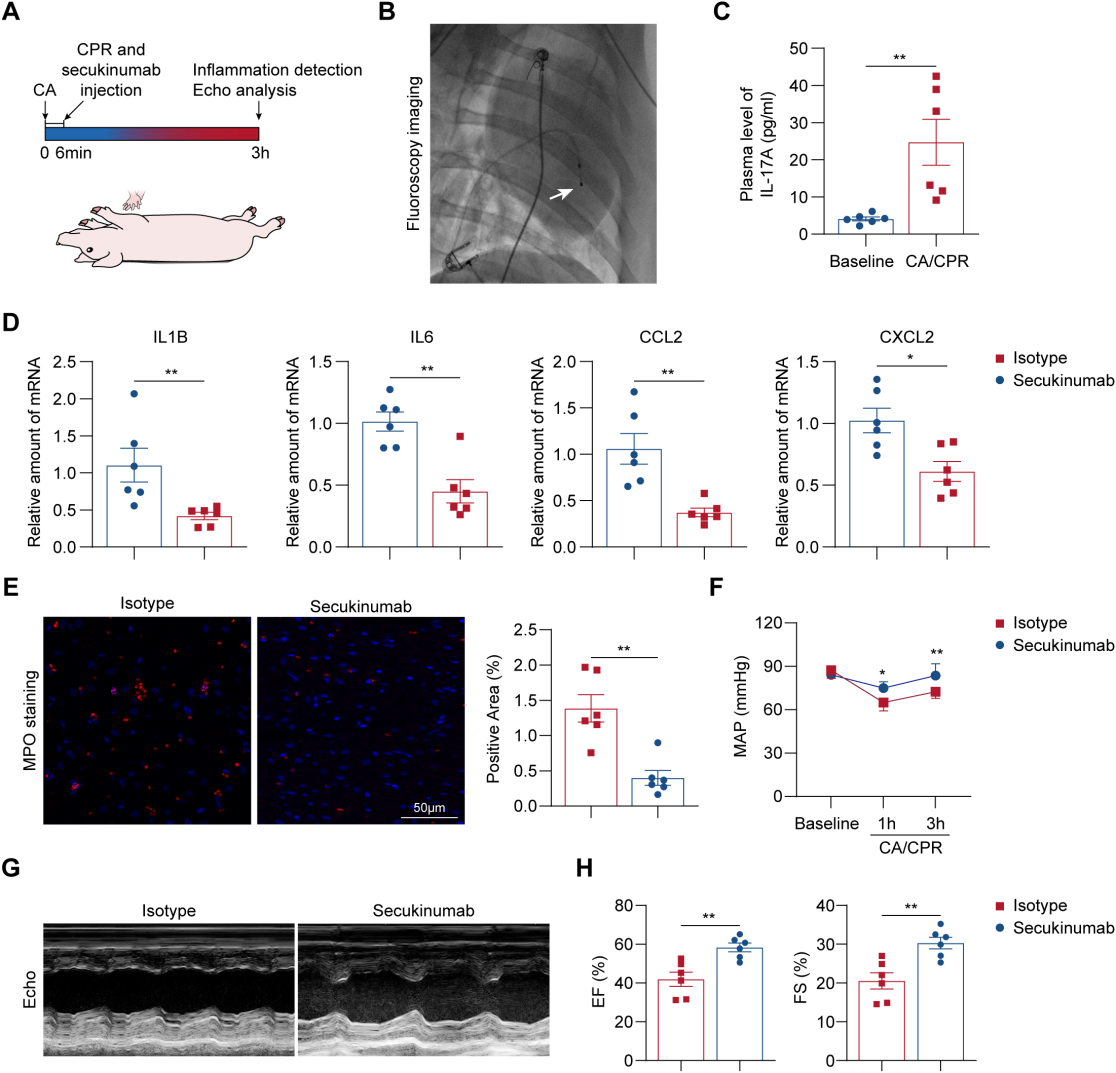
Early inhibition of IL-17A suppresses myocardial inflammation and improves cardiac function in a porcine CA/CPR model. **(A)** Schematic representation of the experiment design in pigs. **(B)** Fluoroscopy image confirmation of successful occupation of temporary pacemaker lead into right ventricular chamber. White arrows indicated temporary pacemaker lead. **(C)** Plasma levels of IL-17A in baseline and 3 h post-resuscitation in porcine model of CA/CPR. (n = 4). Data were analyzed by Student *t* test. **P < 0.01. **(D)** Relative mRNA expression of inflammatory genes in indicated groups after 3 h post-CA/CPR (n = 6). Data were analyzed by Student *t* test. *P < 0.05; **P < 0.01. **(E)** Left panel: Representative immunofluorescence images of MPO staining in indicated groups after 3 h post-CA/CPR (n = 4, scale bar = 50 μm). Right panel: Quantification of immunofluorescence staining presented in the left panel (n = 6). Data were analyzed by Student *t* test. **P < 0.01. **(F)** MAP in indicated groups. Data were analyzed by 2-way ANOVA followed by the Bonferroni *post hoc* test. *P < 0.05; **P < 0.01. **(G)** Representative M-mode echocardiograms of pigs in indicated groups after 3 h post-CA/CPR. **(H)** Quantification of ejection fraction (EF) and fractional shortening (FS) in indicated groups (n = 6). Data were analyzed by Student *t* test. **P < 0.01. CA indicates cardiac arrest; CPR, cardiopulmonary resuscitation; IL-17, interleukin-17; MAP, mean arterial pressure; MPO, myeloperoxidase.

Based on the preceding results, we next investigated whether IL-17A inhibition could alleviate cardiac dysfunction in a preclinical porcine model of CA/CPR. Swine were subjected to ventricular fibrillation induced by a pacing catheter, followed by 6 min of untreated CA (**Figures 5A, B**). CPR was then initiated and secukinumab was administered at the onset of CPR (**Figure 5A**). Our results showed that serum level of IL-17A was dramatically elevated after CA/CPR (**Figure 5C**). The early inhibition of IL-17A by secukinumab significantly attenuated myocardial inflammation, as evidenced by reduced expression of proinflammatory cytokines and chemokines, including *IL1B*, *IL6*, *CCL2*, and *CXCL2* (**Figure 5D**). Consistently, immunofluorescence staining for myeloperoxidase (MPO) demonstrated decreased neutrophil infiltration in the myocardium of secukinumab-treated animals compared with isotype controls (**Figure 5E**). Furthermore, hemodynamic monitoring showed an elevated mean arterial pressure following IL-17A inhibition post-resuscitation (**Figure 5F**). Echocardiographic assessment revealed significant improvement in cardiac function in secukinumab-treated group (**Figures 5G, H**). Together, these results indicate that early pharmacological inhibition of IL-17A ameliorates post-resuscitation myocardial inflammation and improves cardiac functional recovery in a porcine model of CA/CPR.

### 3.6 Early inhibition of IL-17A mitigates brain injury and improves neurological outcomes in a porcine CA/CPR model

**Figure 6:**
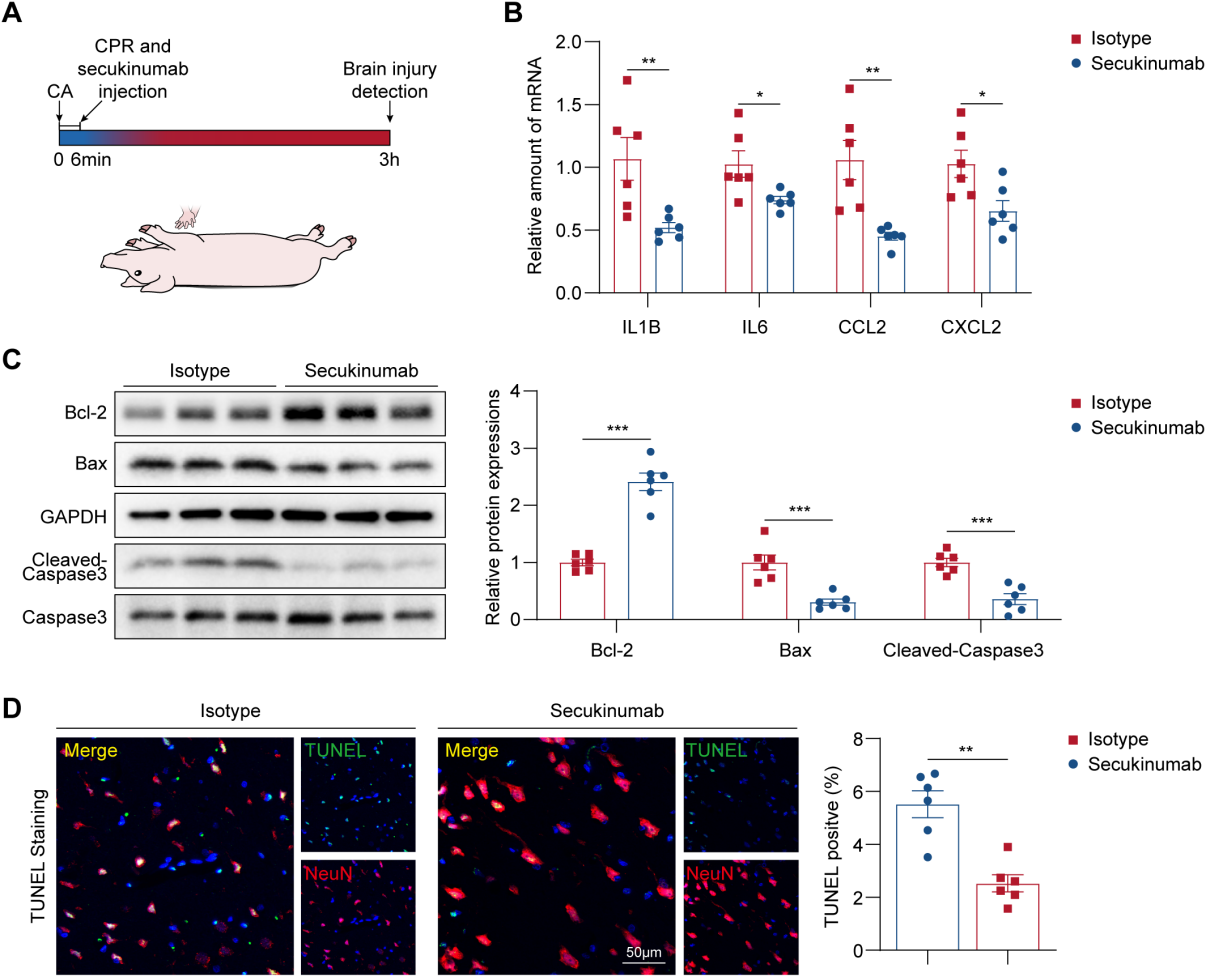
Early inhibition of IL-17A mitigates brain injury and improves neurological outcomes in a porcine CA/CPR model. **(A)** Schematic representation of the experiment design in pigs. **(B)** Relative mRNA expression of inflammatory genes in indicated groups after 3 h post-CA/CPR (n = 6). Data were analyzed by Student *t* test. *P < 0.05; **P < 0.01. **(C)** Left panel: Expression levels of apoptosis-related proteins in cerebral cortex from indicated groups after 3 h post-CA/CPR (n = 6). Right panel: Quantification of protein expressions in indicated groups. Data were analyzed by Student *t* test. ***P < 0.001. **(D)** Left panel: Representative images of neuron apoptosis in cerebral cortex were visualized by TUNEL staining (n = 6, scale bar = 50 μm). Right panel: Quantification of immunofluorescence staining presented in the left panel (n = 4). Data were analyzed by Student *t* test. **P < 0.01. CA indicates cardiac arrest; CPR, cardiopulmonary resuscitation; IL-17, interleukin-17; TUNEL, terminal deoxynucleotidyl transferase dUTP nick-end labeling.

To investigate whether early inhibition of IL-17A can ameliorate neurological injury following CA/CPR, we evaluated inflammatory and apoptotic responses in a porcine model (**Figure 6A**). As expected, secukinumab treatment significantly attenuated neuroinflammation compared with isotype control, as evidenced by decreased expression of pro-inflammatory chemokines including *IL1B*, *IL6*, *CCL2*, and *CXCL2* (**Figure 6B**). We next assessed neuronal apoptosis to determine the neuroprotective potential of IL-17A inhibition. Secukinumab administration in porcine model significantly alleviated apoptosis, indicated by downregulated cleaved caspase-3 and Bax and upregulated expression of Bcl-2 (**Figure 6C**). TUNEL staining further confirmed a reduction in neuronal apoptosis following IL-17A blockade (**Figure 6D**). Collectively, these findings demonstrate that early inhibition of IL-17A suppresses neuroinflammation and attenuates neuronal apoptosis, indicating a neurological recovery in a clinically relevant porcine CA/CPR model.

## 4 Discussion

**Figure 7:**
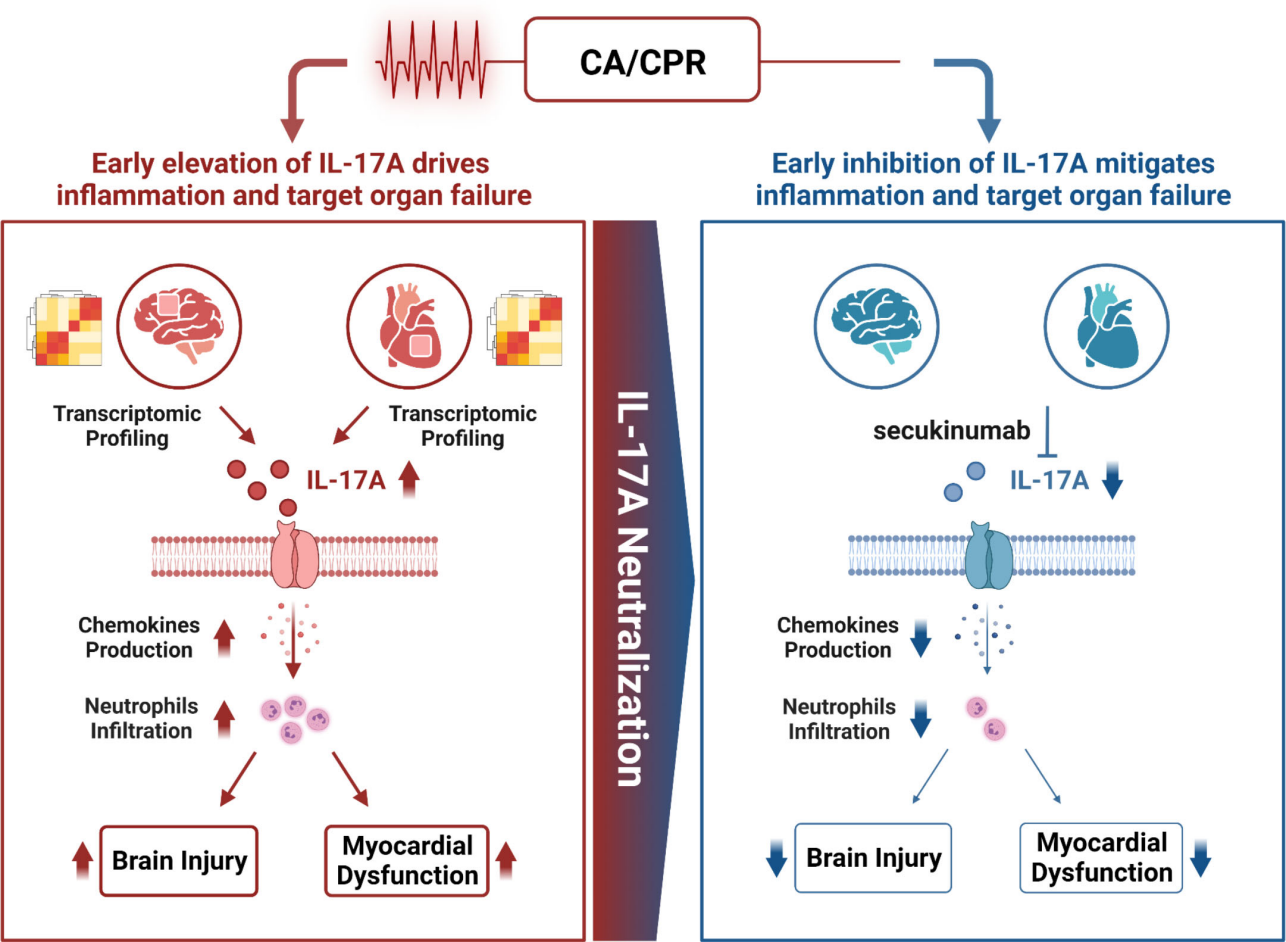
Proposed model for IL-17A in the pathogenesis of CA/CPR.

In the present study, through comprehensive analysis of transcriptomic profiles from cardiac and cerebral tissues at early stages post-CA/CPR, we identified that early inflammatory responses, especially activation of IL-17 signaling pathway, as a key driver of post-PCAS including myocardial dysfunction and brain injury after resuscitation. Early pharmacological inhibition of IL-17A with secukinumab at the initiation of CPR attenuated cardiac inflammation and dysfunction and subsequently alleviated sustained neuroinflammatory injury in both murine and porcine CA/CPR models. Our data provides direct evidence that early activation of IL-17 pathway induces cardiac and cerebral damage, highlighting IL-17A as a promising therapeutic target for promoting multi-systemic inflammatory outcomes following CA (**Figure 7**).

Importantly, inflammation-mediated organ crosstalk plays a critical role in PCAS. Myocardial dysfunction and hemodynamic instability after resuscitation contribute to secondary brain injury, while neuroinflammation further amplifies systemic inflammatory responses and cardiovascular dysfunction (40). This bidirectional interaction between the heart and brain forms a vicious cycle that accelerates multi-organ injury after CA (41). Therefore, identifying early inflammatory mediators that drive this heart–brain inflammatory axis may provide novel intervention strategies for preventing post-resuscitation chronic inflammation and multi-organ injury.

Myocardial dysfunction following CA/CPR is characterized by transient but severe abnormalities of contraction and relaxation of myocardium (42). This malfunction, also termed as “myocardial stunning”, is considered to be a reversible alteration post-resuscitation with nearly full recovery within 72 hours (43–45). Whereas brain injuries post-resuscitation, neuronal apoptosis in particular, may result in sustained neuronal damage and contribute to mortality (39, 46). Notably, the acute changes of cardiac function induced by CA result in global hemodynamic instability and cause additional chronic brain insult (8, 47). However, the pathophysiological process of myocardial dysfunction and brain injury post-resuscitation has not been fully elucidated. Our transcriptomic results demonstrated that CA/CPR-induced elevated inflammatory signature in both heart and brain at early stage of post-resuscitation was closely associated with the following myocardial dysfunction and neuronal damage. Inflammatory response is a critical biological process that has been implicated in both the onset of CA (48, 49) and post-CA/CPR period (11, 50). Studies suggested that patients with higher level of cytokines were at high risk of sudden cardiac death after myocardial infarction (48). Persistent release of cytokines in successfully resuscitated patients after CA has been shown to be associated with poor prognosis (11), a “sepsis-like” syndrome with multi-organ failure including cardiac dysfunction and brain injury (51, 52). These findings indicate that persistent inflammation is a key process driving multi-organ injury after CA/CPR, and that early intervention to prevent the progression of chronic inflammatory responses may represent a critical strategy to improve outcomes after CA.

IL-17A, generated by IL-17-producing cells such as T helper 17 (Th17) cells, γδT cells, and natural killer T (NKT) cells, has been shown to play critical roles in inflammatory diseases, allergic diseases and autoimmune disorders (53–55). IL-17A serves as a proinflammatory cytokine that can trigger the expressions of chemokines, leading to neutrophils infiltration in target organs (56, 57). Recently, evidence shows that IL-17A could directly mediate cardiomyocyte apoptosis in cardiac ischemia/reperfusion injury (58) and induce detrimental effects during acute ischemic stroke (59, 60). Previous studies have also implicated IL-17A signaling in CA, particularly in mediating neuroinflammatory responses and neurological injury following ischemic insult, thereby contributing to post-resuscitation brain damage (61, 62). These findings further support a role for IL-17A in post-CA pathophysiology and provide context for our results. In addition to its role in inflammation, accumulating evidence suggests that IL-17A is involved in the pathogenesis of chronic inflammatory diseases, including atherosclerosis, cardiac remodeling, heart failure, ischemic stroke, and neurodegenerative diseases, where persistent IL-17 signaling contributes to chronic inflammation, tissue remodeling, and organ dysfunction (63–66). In the present study, by delineating the transcriptome changes of two critical organs involved in PCAS, our findings demonstrated that IL-17 signaling pathway was dramatically activated (ranked top 1 in the heart and top 2 in the brain), leading to generation of chemokines and interleukins and later neutrophils infiltration during early post-resuscitation period. Investigation of serum IL-17 isoforms revealed IL-17A as a critical cytokine with highest fold change of production among IL-17 family members in post-resuscitation condition. Notably, we found that survivors who underwent sudden CA resulted from STEMI showed concomitant elevation of plasma level of IL-17A compared with their non-CA PSM-matched controls in post-resuscitation period, concomitant with the observations in non-MI CA populations (67). However, given the limited sample size and single time-point measurement, these findings should be interpreted with caution and require validation in larger prospective studies. Collectively, our results indicate that early activation of IL-17A signaling is a key inflammatory driver of multi-organ failure post-resuscitation.

An important question arising from our findings concerns the cellular source of IL-17A in the post-resuscitation period. The rapid elevation of serum IL-17A within 3 hours of CA/CPR suggests that the conventional adaptive immune response, which typically requires days for T helper 17 (Th17) cell differentiation and expansion, is unlikely to be the primary driver of early IL-17A production. Instead, innate-like lymphocytes, including γδT cells, natural killer T (NKT) cells, and group 3 innate lymphoid cells (ILC3s), are capable of rapidly producing IL-17A upon activation by damage-associated molecular patterns (DAMPs) and pro-inflammatory cytokines such as IL-1β and IL-23, which are known to be elevated after global ischemia/reperfusion injury. Indeed, γδT cells have been identified as early responders producing IL-17A in myocardial ischemia/reperfusion injury and ischemic stroke. Furthermore, neutrophils themselves may serve as an additional source of IL-17A under certain inflammatory conditions, potentially creating a positive feedback loop that amplifies the inflammatory cascade. Future studies utilizing flow cytometry, single-cell RNA sequencing, or conditional knockout models will be essential to delineate the cellular hierarchy of IL-17A production in the post-CA setting. Mechanistically, IL-17A is known to exert its proinflammatory effects through binding to the IL-17 receptor complex (IL-17RA/IL-17RC), which recruits the adaptor protein Act1 (also known as CIKS) to activate the TRAF6/NF-κB and MAPK signaling cascades, ultimately driving the transcription of chemokines (CXCL1, CXCL2, CCL2) and pro-inflammatory cytokines (IL-1β, IL-6). Our transcriptomic data showed significant upregulation of NF-κB signaling pathway components in both heart and brain tissues after CA/CPR (**Figures 1I**, **2C**), suggesting that the Act1/TRAF6/NF-κB axis may represent the downstream effector pathway of IL-17A in this context. The attenuation of chemokine expression and neutrophil infiltration following secukinumab treatment is consistent with blockade of this signaling cascade. However, direct demonstration of this mechanistic link through interrogation of specific downstream signaling nodes (e.g., phosphorylated NF-κB p65, TRAF6 expression) was not performed in the current study and warrants future investigation.

Anti-IL-17A therapy has emerged as a potential therapeutic management in both chronic cardiovascular and neurological diseases. In the cardiovascular system, neutralization of IL-17A has been shown to confer protection against atherosclerotic lesion progression and myocardial ischemia-reperfusion injury (68). In the central nervous system, inhibition of IL-17A signaling alleviates amyloid-β–induced neuroinflammation and memory impairment in Alzheimer’s disease (69) and reduces blood-brain barrier disruption and dopaminergic neurodegeneration in Parkinson’s disease (70). However, anti-IL-17A therapy raised concerns for its long-term use, particularly in the context of chronic inflammatory states, as observed in infection (71) and adverse cardiac events (72), suggesting that the timing and disease setting of IL-17A modulation may be critical determinants of its therapeutic benefit. Nevertheless, the role of IL-17A-targeted intervention in the setting of CA, particularly in preclinical CA/CPR models, remains unclear. Importantly, our findings highlight a hyper-acute inflammatory response following CA, with IL-17 signaling rapidly activated during the early post-resuscitation period. Notably, IL-17A inhibition was only effective when administered at the initiation of CPR, whereas delayed administration failed to confer survival benefit. These results suggest the existence of a narrow therapeutic window and underscore the importance of ultra-early intervention, potentially in the pre-hospital setting, to effectively mitigate post-CA inflammation and multi-organ injury. Of note, while the IMICA trial demonstrated that IL-6 receptor blockade with tocilizumab could attenuate the systemic inflammatory response after out-of-hospital cardiac arrest (11), our findings suggest that IL-17A may function as an upstream orchestrator of the inflammatory cascade, driving the production of multiple downstream mediators including IL-6. Targeting IL-17A may therefore provide broader suppression of the post-resuscitation inflammatory response, limit multi-organ failure following resuscitation, thereby improving survival after CA. Whether continued IL-17A blockade is necessary during subsequent chronic inflammation requires further experimental exploration and clinical evidence.

## 5 Limitations

Several limitations of the present study should be acknowledged. First, although our transcriptomic and functional data demonstrate early activation and sustained expression of IL-17 signaling pathway genes in brain tissues up to 72 hours post-resuscitation, the observation window of this study was confined to the acute and subacute phases (0–72 h). Whether the inflammatory response driven by IL-17A evolves into a bona fide chronic inflammatory state beyond this time frame remains to be determined. Future studies with extended observation periods (7–30 days) are warranted to characterize the long-term trajectory of IL-17A-mediated inflammation and its contribution to chronic organ dysfunction after CA. Second, the murine CA/CPR model employed potassium chloride-induced asystole, which differs from the clinical scenario where CA is predominantly caused by ventricular fibrillation or pulseless electrical activity. Although the porcine model utilized ventricular fibrillation-induced CA, which more closely recapitulates the clinical setting, the translational relevance of our findings should be interpreted with this model-specific limitation in consideration. Third, the cellular source(s) of IL-17A following CA/CPR were not identified in this study. While IL-17A is classically produced by T helper 17 (Th17) cells, the rapid elevation of IL-17A within 3 hours post-resuscitation suggests that innate immune cells, such as γδT cells, natural killer T (NKT) cells, or group 3 innate lymphoid cells (ILC3s), may serve as the primary early source of IL-17A in this context. Characterizing the cellular origin of IL-17A in post-CA inflammation represents an important direction for future mechanistic studies. Fourth, secukinumab was administered at the initiation of CPR, a time point that poses practical challenges in clinical settings, particularly in out-of-hospital cardiac arrest scenarios where immediate access to biologic agents is limited. Moreover, delayed administration of secukinumab at 3 or 24 hours post-resuscitation failed to improve survival (**Supplementary Figures 3A, B**), suggesting a narrow therapeutic window that warrants further investigation to determine the latest effective time point for intervention. Future studies should explore alternative delivery strategies, such as pre-hospital administration protocols or fast-acting small-molecule IL-17A inhibitors, to enhance clinical feasibility. Fifth, the downstream signaling mechanism through which IL-17A drives multi-organ injury after CA/CPR was not elucidated. IL-17A is known to activate the Act1/TRAF6/NF-κB signaling axis in various inflammatory contexts, and whether this canonical pathway mediates the post-resuscitation inflammatory cascade requires further investigation. Sixth, the human clinical data in this study were derived from a retrospective analysis of 26 propensity score-matched pairs of STEMI patients with or without CA, with IL-17A measured at a single time point. This limited sample size and single-time-point design preclude the establishment of a causal relationship between IL-17A elevation and clinical outcomes. Larger prospective cohort studies with serial IL-17A measurements and correlation analyses with neurological and cardiac functional outcomes are needed to validate the clinical relevance of our findings. Finally, the porcine model did not include long-term survival analysis or neurological functional scoring, which limits the assessment of the sustained neuroprotective effects of IL-17A inhibition in a clinically relevant large animal model. Additionally, RNA-seq analysis was performed with three biological replicates per group, which may have limited statistical power to detect genes with moderate effect sizes, although key findings were independently validated by RT-qPCR in a larger sample set (n = 6).

## 6 Conclusions

Our current experimental findings are scientifically and clinically important. By utilization of both murine and porcine model of CA/CPR, we have identified the IL-17 signaling pathway as a key driver of inflammation response during the early post-resuscitation period. This novel insight into the pathogenesis of CA/CPR might broaden our current understanding of this life-threatening condition. Importantly, we have showed that early inhibition of IL-17A may serve as a prospective therapeutic strategy for the intervention of CA and subsequent multi-organ failure.

## Data Availability

The datasets are publicly available. The accession numbers are as follows: BioProject accession numbers PRJNA1441882 and PRJNA1441828. The raw sequencing data are available in the NCBI Sequence Read Archive (SRA). Links: https://www.ncbi.nlm.nih.gov/bioproject/?term=PRJNA1441882, https://www.ncbi.nlm.nih.gov/bioproject/?term=PRJNA1441828.

## Glossary

CA/CPR: Cardiac arrest/cardiopulmonary resuscitation
IL-17A: Interleukin-17A
PCAS: Post cardiac arrest syndrome
STEMI: ST-elevation myocardial infarction
ROSC: Return of spontaneous circulation
PSM: Propensity score-matched analysis
TCC: Traditional chest compression
NCC: Novel chest compression
BPM: Beats per minute
MAP: Mean arterial pressure
EF: Ejection fraction
FS: Fractional shortening
IVRT: Isovolumic relaxation time
DEGs: Differentially expressed genes
PCA: Principal components analysis
Th17: T helper 17
NKT: Natural killer T
TUNEL: Terminal deoxynucleotidyl transferase dUTP nick-end labeling
