# Supplementary material for: Systemic inflammatory response drives post-cardiac arrest multi-organ injury via IL-17 signaling in murine and porcine models
Source: Front Immunol. 2026 Apr 29;17:1837813. doi: 10.3389/fimmu.2026.1837813 (PMC13167478; doi:10.3389/fimmu.2026.1837813)
Supplement: Supplementary file 1 [file Table1.doc]

**SUPPLEMENTARY MATERIAL**

**Systemic Inflammatory Response Drives Post-Cardiac Arrest Multi-Organ Injury via IL-17 Signaling in Murine and Porcine Models**

Yang Yan^1#^, Taiwei Chen^1#^, Ancai Yuan^1#^, Na Geng^1^, Fang Wan^1^, Peiliang Fang^1^, Zhiqing Qiao^1^, Zhaoling Wei^1^, Jun Pu^1,2*^

^1^Department of Cardiology, Ren Ji Hospital, School of Medicine, Shanghai Jiao Tong University; State Key Laboratory for Oncogenes and Related Genes, Shanghai Cancer Institute, Shanghai Jiao Tong University, Shanghai 200120, China.

^#^These authors contributed equally to this work

^*^Address correspondence to:

Jun Pu, MD, Ph.D., FAHA, FESC

Department of Cardiology, Ren Ji Hospital, School of Medicine, Shanghai Jiao Tong University; State Key Laboratory for Oncogenes and Related Genes, Shanghai Cancer Institute, Shanghai Jiao Tong University, Shanghai, China. E-mail: pujun310@hotmail.com

**Table S1 Survivors with sudden CA who underwent CPR versus their non-CA/CPR PSM-matched AMI patients.**

|  | Non-CA/CPR  (n=26) | CA/CPR  (n=26) | *P*-value |
| --- | --- | --- | --- |
| Age (yrs) | 58.00 (49.5-62.00) | 55.50 (50.75-66.50) | 0.735 |
| Male gender (n, %) | 23 (88.5) | 25 (96.2) | 0.610 |
| Body mass index (kg/m^2^) | 24.62 ± 2.93 | 25.23 ± 3.14 | 0.466 |
| Medical history (n, %) |  |  |  |
| Smoking | 20 (76.9) | 20 (76.9) | 1.000 |
| Hypertension | 14 (53.8) | 12 (46.2) | 0.782 |
| Diabetes | 9 (34.6) | 5 (19.2) | 0.349 |
| Hyperlipidemia | 12 (46.2) | 10 (38.5) | 0.779 |
| Renal dysfunction | 0 (0) | 1 (3.8) | 1.000 |
| Killip class (n, %) |  |  | 1.000 |
| I | 15 (57.7) | 16 (61.5) |  |
| II or higher | 11 (42.3) | 10 (38.5) |  |
| Culprit vessel (n, %) |  |  | 0.245 |
| LAD | 18 (69.2) | 14 (53.8) |  |
| LCX | 1 (3.8) | 0 (0) |  |
| RCA | 7 (26.9) | 12 (46.2) |  |
| TNI (ng/mL) | 26.24 (2.08–51.87) | 20.93 (11.76–102.00) | 0.358 |
| BNP (pg/mL) | 100.95 (33.60–398.75) | 115.00 (20.7–436.25) | 0.862 |
| RBC (10^12^/L) | 4.51 ± 0.39 | 4.61 ± 0.38 | 0.369 |
| Hemoglobin (g/L) | 141.64 ± 11.64 | 144.65 ± 10.06 | 0.328 |
| WBC (10^9^/L) | 11.68 (10.09–13.45) | 13.67 (10.71–15.96) | 0.079 |
| D-dimer (mg/L) | 0.15 (0.10-0.30) | 0.18 (0.14-0.35) | 0.296 |
| FBG (mmol/L) | 5.57 (4.98-5.97) | 5.84 (5.21-6.31) | 0.207 |
| TG (mmol/L) | 1.29 (0.91-2.05) | 1.23 (0.99-1.76) | 0.877 |
| TC (mmol/L) | 4.77 ± 1.10 | 4.82 ± 1.01 | 0.864 |
| HDL (mmol/L) | 1.12 ± 0.18 | 1.10 ± 0.18 | 0.636 |
| LDL (mmol/L) | 3.16 ± 0.90 | 3.03 ± 0.67 | 0.549 |
| SCr (μmol/L) | 67.15 ± 12.24 | 81.85 ± 19.07 | 0.002 |
| ALT (U/L) | 53.00 (22.75-72.35) | 57.00 (41.00-87.00) | 0.022 |

Study patient data were obtained from the resources of the EARLY-MYO-CMR (Early Assessment of Myocardial Tissue Characteristics by CMR in STEMI) registry, which was a prospective, multicenter registry of patients with acute ST-elevation myocardial infarction (STEMI) who have undergone CMR imaging (NCT03768453). Data are shown as mean ± SD, median (IQR) or n (%). 52 study participants (26 CA/CPR patients and 26 matched control subjects) were recruited. Comparisons of continuous variables were performed by Student’s *t*-test for normally-distributed variables or the Mann–Whitney *U* test for variables that were not normally distributed. Comparisons of categorical variables were conducted with chi-square test or Fisher’s exact test. Abbreviations: TNI, troponin I; BNP, brain natriuretic peptide; RBC, red blood cell; WBC, white blood cell; FBG, fasting blood glucose; TG, triglyceride; TC, total cholesterol; HDL, high-density lipoprotein; LDL, low-density lipoprotein; SCr, serum creatinine; ALT, alanine aminotransferase.

**Table S2 Primers for real-time quantitative PCR**

| **Gene name** | **Primer** | **Sequence** |
| --- | --- | --- |
| *Actb* | Forward 5’-3’ | CCGTGAAAAGATGACCCAGA |
|  | Reverse 5’-3’ | TACGACCAGAGGCATACAG |
| *Cxcl1* | Forward 5’-3’ | CCGAAGTCATAGCCACACTCAA |
|  | Reverse 5’-3’ | CAAGGGAGCTTCAGGGTCAA |
| *Cxcl2* | Forward 5’-3’ | TGACTTCAAGAACATCCAGAGCTT |
|  | Reverse 5’-3’ | CTTGAGAGTGGCTATGACTTCTGTCT |
| *Cxcl10* | Forward 5’-3’ | CCAAGTGCTGCCGTCATTTTC |
|  | Reverse 5’-3’ | GGCTCGCAGGGATGATTTCAA |
| *Ccl2* | Forward 5’-3’ | TTAAAAACCTGGATCGGAACCAA |
|  | Reverse 5’-3’ | GCATTAGCTTCAGATTTACGGGT |
| *Ccl7* | Forward 5’-3’ | GCTGCTTTCAGCATCCAAGTG |
|  | Reverse 5’-3’ | CCAGGGACACCGACTACTG |
| *Ccl12* | Forward 5’-3’ | ATTTCCACACTTCTATGCCTCCT |
|  | Reverse 5’-3’ | ATCCAGTATGGTCCTGAAGATCA |
| *Il1β* | Forward 5’-3’ | TCGCAGCAGCACATCAACAAGAG |
|  | Reverse 5’-3’ | AGGTCCACGGGAAAGACACAGG |
| *Il6* | Forward 5’-3’ | GAGGATACCACTCCCAACAGACC |
|  | Reverse 5’-3’ | AAGTGCATCATCGTTGTTCATACA |


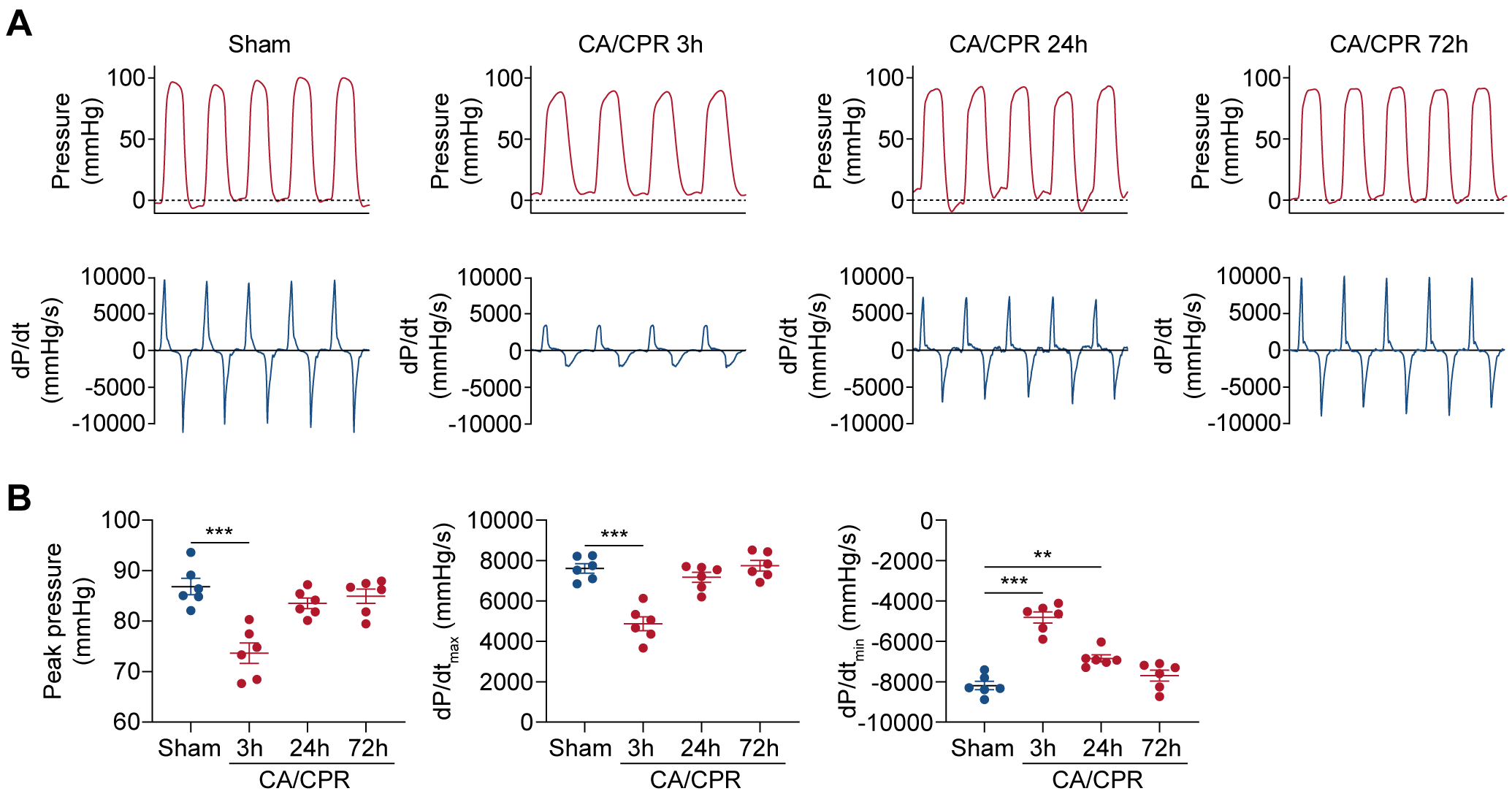


**Fig. S1. Hemodynamic monitoring in CA/CPR murine model.**

(**A**, **B**) Quantification of peak pressure, dP/dt_max_ and dP/dt_min_ in indicated groups (n = 6). Data were analyzed by one-way ANOVA followed by Bonferroni’s post hoc test. ***P*<0.01; ****P*<0.001.


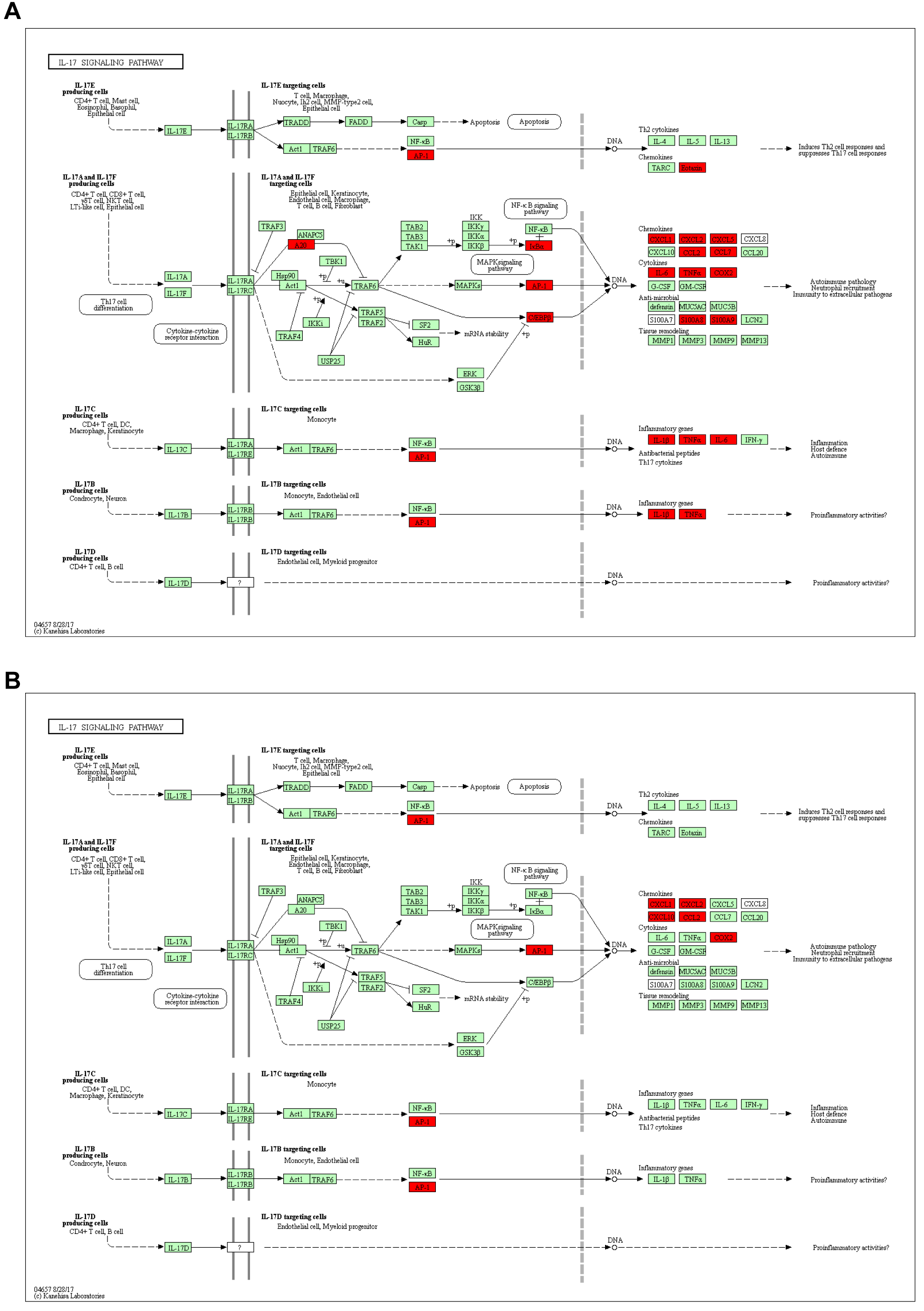


**Fig. S2 Changes of IL-17 signaling pathway post-resuscitation.**

(**A**) Pathway map of notable DEGs in heart as determined using the KEGG database. (**B**) Pathway map of notable DEGs in brain as determined using the KEGG database.


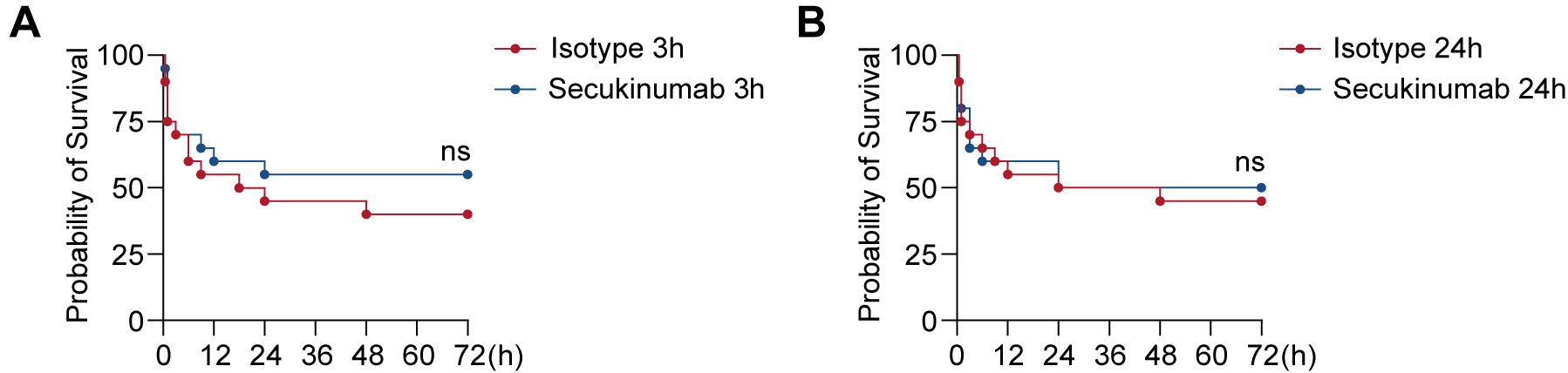


**Fig. S3 The impact of late IL-17A inhibition on survival rates.**

(**A**, **B**) Survival curves in indicated groups (n = 20 per group). The administration of secukinumab or isotype control were performed in 3 h (A) or 24 h (B) post-CA/CPR period. Survival data were analyzed by the Kaplan-Meier method and compared using log-rank tests. ns = not significant.


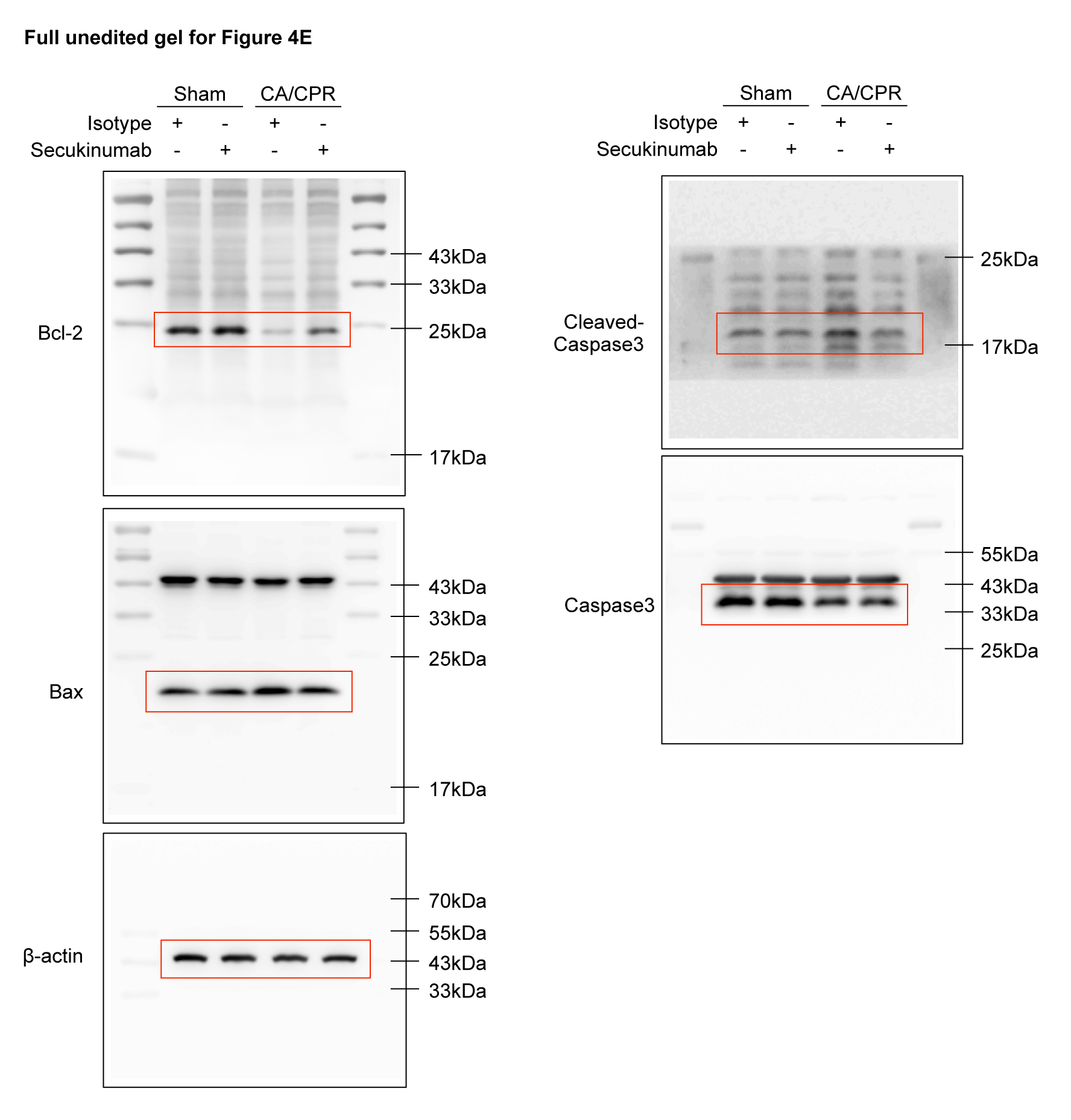


**Fig. S4 Full unedited gel for Figure 4E.**


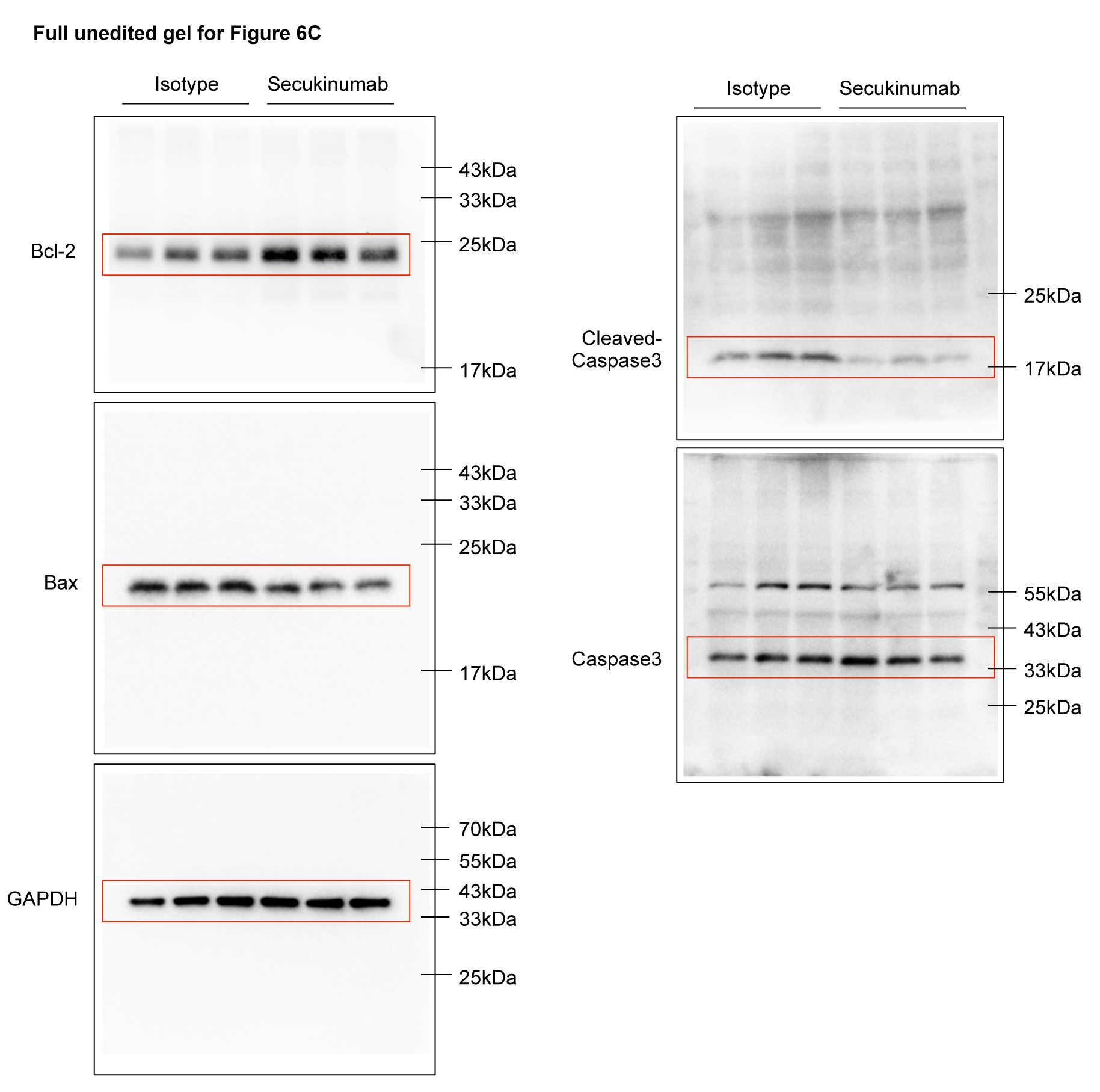


**Fig. S5 Full unedited gel for Figure 6C.**
